# Perspectives on Fetal Sex and Prenatal Diagnosis of Differences of Sex Development Among Midwives

**DOI:** 10.1002/pd.6889

**Published:** 2025-09-15

**Authors:** Madeline Dingle, Sharon Aufox, Emilie K. Johnson, Jeffrey S. Dungan, Aimee Clark, Katie Abihider, Allison Goetsch Weisman

**Affiliations:** ^1^ Graduate Program in Genetic Counseling Northwestern University Chicago Illinois USA; ^2^ Center for Genetic Medicine Northwestern University Feinberg School of Medicine Chicago Illinois USA; ^3^ Division of Urology Ann & Robert H. Lurie Children's Hospital of Chicago Chicago Illinois USA; ^4^ Department of Urology Northwestern University Feinberg School of Medicine Chicago Illinois USA; ^5^ Department of Obstetrics and Gynecology Northwestern University Feinberg School of Medicine Chicago Illinois USA; ^6^ Division of Clinical Genetics Department of Obstetrics and Gynecology Northwestern University Feinberg School of Medicine Chicago Illinois USA; ^7^ Division of Endocrinology Ann & Robert H. Lurie Children's Hospital of Chicago Chicago Illinois USA; ^8^ Division of Genetics Genomics & Metabolism Ann & Robert H. Lurie Children's Hospital of Chicago Chicago Illinois USA; ^9^ Department of Pediatrics Northwestern University Feinberg School of Medicine Chicago Illinois USA

## Abstract

**Objective:**

Cell‐free DNA screening has increased prenatal diagnosis/suspicion of fetal differences of sex development (DSD). This study explored how midwives discuss fetal sex and possible DSD with pregnant patients.

**Method:**

Active members of the American College of Nurse‐Midwives were surveyed electronically to assess terminology use when discussing fetal sex and DSD, terminology influences, and comfort and preparedness levels when discussing a fetal DSD.

**Results:**

Most participants (59.1%) reported a preference for use of gender‐specific language (e.g., baby boy or baby girl) when disclosing predicted fetal sex results, often reflecting language used by patients. However, use of non‐gendered language increased (40.9% vs. 76.2%) with suspicion of fetal DSD. Participants reported discomfort (41.9%) and unpreparedness (48.1%) when disclosing results related to genital variation. Additionally, some participants reported comfort with using terminology that may be inappropriate or outdated when discussing DSD (e.g., hermaphrodite, 16%). While a minority of participants (20.3%) reported having prior education about DSD, most participants (81.7%) desired education on this topic. Notably, participants with prior DSD education consistently expressed higher levels of preparedness for discussing DSD compared with those who did not receive this education.

**Conclusion:**

Findings underscore the importance of DSD education for midwives to increase knowledge, comfort, and preparedness in this area of care.

## Highlights

- What's already known about this topic?
  ◦ cfDNA has led to more prenatal detection of differences of sex development (DSD).
  ◦ Patients report confusion surrounding a prenatal DSD finding.
  ◦ Education about fetal sex/DSD is lacking in prenatal clinicians' education.
- What does this study add?
  ◦ Midwives use gendered language to discuss fetal sex, and sex‐based language for DSD.
  ◦ Some midwives use terms to refer to DSD that are outdated/inappropriate.
  ◦ Midwives report little to no formal training on DSD and desire additional education.

## 1 Introduction

The term “sex” is a biological construct based on anatomy, genetics, and hormones, while the term “gender” is a social construct that includes gender identity, expression, and societal expectations regarding the characteristics and behaviors linked to biological sexes [1, 2]. Despite sex and gender being two unique concepts, these terms are regularly used interchangeably in the United States. Furthermore, in pregnancies, predicted fetal sex is often assumed to communicate gender [3, 4, 5, 6]. Incorrectly using these terms continues to blur their respective meanings. This reinforces the assumption among patients that sex and gender are interchangeable, an individual can't identify as intersex or associate themselves with one, none, or several genders, and that gender and sex are completely unrelated [3, 4].

Although, sex and gender are distinct entities, they are not unassociated. Past literature has shown how these concepts are separate but still dependent upon one another [4, 7]. Gender identity is more often tied to societal expectations–masculinized and feminized traits–that are associated with sex [2, 8]. An unclear understanding of how sex and gender differ and relate to one another reinforces gender essentialism—the belief that gender is a fixed binary attribute tied to assigned biological sex. By conflating these concepts, the belief that gender identity is preset by one's assigned sex at birth is continuously reinforced. This perpetuation of a rigid framework around sex and gender has led to the exclusion and pathologization of individuals who do not conform to binary notions of sex and gender within the medical community [6, 7].

Both treating sex and gender as if they are unrelated and using the terms interchangeably reinforce preexisting modes for how these terms are misused. For example, assigning a gender at birth that has erroneously been correlated with either male or female sets up the potential for a child to experience gender dysphoria due to not falling within the expectations of their baselessly assigned gender [9, 10, 11]. However, assigning a gender to a fetus in the prenatal period has become a popular practice to help expectant parents bond with the future baby, prepare for the birth date, or to connect family and friends to the pregnancy (e.g., through a “gender reveal” party) [6, 12].

The discovery of predicted fetal sex has inadvertently become easier for many expectant parents through advancements in fetal aneuploidy screening. Cell‐free DNA (cfDNA) screening technology, previously referred to as non‐invasive prenatal screening (NIPS), was first made available to pregnant patients as a fetal chromosomal aneuploidy screen in the United States in 2011 [13, 14]. The American College of Obstetrics and Gynecology (ACOG) recommends the use of cfDNA screening technology for chromosomal aneuploidy screening, including sex chromosome aneuploidies (SCA). This type of screening can identify the presence or absence of Y chromosome material, which is predictive of fetal chromosomal sex [15]. It has become common for many consumers and some media sources to incorrectly refer to cfDNA screening technology as “the gender test” as many pregnant patients pursue this option to learn predicted fetal sex earlier in pregnancy than their anatomy scan [9, 12].

In addition to its primary application in identifying common chromosomal aneuploidies, cfDNA screening technology also plays a crucial role in detecting possible differences of sex development (DSD). DSD is a diverse group of diagnoses in which sex chromosomes, hormone levels, internal reproductive anatomy, and/or external genital appearance differ from what's been previously described as “typical” male or female pathways of development [16]. Depending on the breadth of the definition, DSD may occur as frequently as 1 in every 100–200 people [17, 18]. A range of terms has been proposed as alternatives to DSD, for example, variations of sex characteristics (VSC) or intersex (i) [19, 20]. We acknowledge that there is a lack of consensus regarding i/VSC/DSD terminology among healthcare providers and the i/VSC/DSD community and use the term DSD throughout this article as it was the language used to communicate with participants during this research. Additionally, terminology used to describe external genitalia that doesn't align with binary descriptions of male or female development has evolved over time. Although terms such as “genital variation” and “genital variant” are now considered more appropriate in light of recent findings, the term “non‐binary genitalia” was used in the survey at the time it was conducted [18].

The increased use of cfDNA screening has led to more DSD being diagnosed or suspected prenatally [21, 22]. In combination, the use of cfDNA screening and fetal ultrasound imaging typically aids in the detection and subsequent diagnosis of a possible fetal DSD through one of three mechanisms: (1) cfDNA screening results suggestive of an increased risk for a fetal SCA; (2) prenatal ultrasound identifying a variation in the appearance of the fetal genitalia indicative of a likely DSD; and, (3) “genotype‐phenotype discordance” between predicted fetal chromosomal sex (i.e., XX or XY) via cfDNA screening and the appearance of the fetal genitalia on ultrasound [12].

It is common for counseling that precedes prenatal screening to lack clear explanations about the potential to identify a DSD [6, 23, 24]. While it is essential to use thoughtful and respectful language when engaging with the DSD community, it is equally important to recognize that terminology related to anatomical and reproductive development is continually evolving. This evolution is driven by the contributions of community members, researchers, and medical professionals who seek to shift language away from medicalization and pathology toward more affirming, identity‐centered terms [23, 24, 25]. As this language continues to change, both now and in the future, it increasingly reflects the values and perspectives of those within the DSD community. For prenatal clinicians, embracing and staying informed about these changes is vital to offering inclusive, respectful, and affirming care. Lack of awareness of updated terminology may lead to practitioners' use of inaccurate and outdated terminology to describe concepts related to the DSD community [5, 15, 24].

In addition to use of inaccurate or outdated terminology, the conflation of sex and gender can create confusion for pregnant patients about what results indicate, which undermines true informed consent [12, 23, 24]. Consequently, prenatal suspicion or diagnosis of a fetal DSD is associated with pregnant patients experiencing feelings of anxiety, depression, and even suicidal ideation [12, 22]. In today's prenatal care landscape, clinicians have a responsibility to provide accurate counseling and education around prenatal screening while promoting awareness of sex spectrums and gender fluidity [23, 24]. Additionally, it is crucial for prenatal clinicians to be adept at sensitively and accurately disclosing findings suggestive of a possible fetal DSD due to the previously mentioned impact that prenatal screening results concerning a DSD can have on prospective parents.

Certified midwives and certified nurse midwives (CMs/CNMs) are obstetrics providers that have grown in patient utilization over the past 25 years. CMs/CNMs may be preferred by pregnant patients due to their unique ability to provide mental, physical, and emotional care for both the pregnant patient and their child before, during and after pregnancy [26, 27]. This is done by allowing their practice to be guided by the preferences, needs, and values of the patients they care for. Doing so helps build trust, which is widely recognized as a main pillar of the midwifery practice [26, 27]. With this holistic care model, CMs/CNMs provide patient support and guidance that exceeds the clinical space [26, 27]. Given the increased use of cfDNA screening in obstetrics care, it is essential for CMs/CNMs to be trained in offering counseling, resources, and accurate explanations that accompany both expected and unexpected prenatal screening results [28]. This study aimed to assess CMs/CNMs experiences with cfDNA screening results regarding fetal sex prediction and suspicion of a possible fetal DSD, a previously unexplored area. This was done by evaluating the terminology that CMs/CNMs use when talking about fetal sex with patients, identifying what factors influence their terminology choices, and assessing how comfortable and prepared CMs/CNMs are when fetal sex results are atypical or unexpected.

## 2 Methods

### 2.1 Eligibility Criteria

Eligible participants included CMs/CNMs who were at least 18 years of age, currently practicing in the United States, English speaking, and had a work history of at least 1 year. Informed consent was obtained on the landing page of the survey after participants self‐screened for the previously mentioned inclusion criteria. After indicating their agreement to the consent form, participants were able to continue with the survey. If they did not agree or did not meet the eligibility criteria, the individual was able to exit the survey. Participants could exit the survey at any time because the survey and overall participation were voluntary.

### 2.2 Instrumentation

This quantitative study was created through the online survey software REDCap [29, 30] and approved for use by the Northwestern University Institutional Review Board (STU00220287) on November 7th, 2023. The survey was developed based on published literature and authors' expertise and did not employ formal or validated measures. The questions were designed to assess demographic information, terminology that CMs/CNMs prefer to use when discussing fetal sex and suspected DSD, influences on the terminology that CMs/CNMs use to discuss fetal sex and DSD, and preparedness and comfort level when discussing fetal sex and DSD.

### 2.3 Recruitment

An invitation to participate in the study with a link to the survey was sent to members of the American College of Nurse Midwives (ACNM). At the time of distribution, there were a total of 6445 individuals who were registered members of ACNM per the 2019 Core Data Survey. Membership requirements for ACNM include being a CNM, CM, student enrolled in an ACME‐accredited midwifery education program, or an interested professional (American%20College%20of%20Nurse%20Midwives) [31]. The survey was distributed through the organization's biweekly email newsletter to 5389 active ACNM members who are currently practicing. This survey was sent twice: once on December 13th, 2023, and again on December 20th, 2023. Response collection was closed on December 30th, 2023.

### 2.4 Data Analysis

Data from both complete and incomplete surveys were compiled, coded, and analyzed using Microsoft Excel. The threshold for inclusion in the study was answering at least one question in the survey. Submitted surveys with entirely blank responses were not included in the data analysis. Descriptive statistics were calculated for each individual survey question. Categorical variables were outlined by frequency and percentage, while numerical variables were highlighted by mean or median. Chi‐square statistics (or Fisher's exact test) and *p*‐values (*p* < 0.05) were used to compare categorical variables and determine what, if any, statistical significance or associations exist between responses. Answers from incomplete surveys were analyzed and included in the study findings; however, missing data was excluded.

## 3 Results

### 3.1 Survey Response

218 individuals consented to participate, and 177 individuals subsequently completed the survey.

### 3.2 Participant Demographics

As shown in Table 1, the majority of study participants were self‐reported to be cisgender females (*n* = 148; 90.8%), had a background education prior to becoming a midwife as a Bachelor of Science/Registered Nurse (*n* = 111, 62.7%), and had a Certified Nurse Midwife certification (*n* = 173; 91.1%). There was a diverse representation of responses in terms of participant age, years in practice, geographical region, and work setting. Geographical locations were previously determined by ACNM (ACNM regions).

**TABLE 1:** Participants' self‐reported demographic information (*n* = 177).

	*n*	%
Age (years) (*n* = 172)		
26–35	38	22.1
36–45	48	27.9
46–55	38	22.1
56–65	33	19.2
66–79	15	8.7
Time in practice (years) (*n* = 164)		
1–9	80	48.8
10–19	41	25
20–29	28	17.1
30–39	10	6.1
40–49	5	3
Reported gender(s) (*n* = 176)		
Cisgender female	148	90.8
Transgender female	2	1.2
Transgender male	2	1.2
Cisgender male	4	2.5
Genderqueer	5	3.1
Agender	2	1.2
Non‐binary	5	3.1
Questioning	1	0.6
Other	6	3.7
Prefer not to answer	13	90.8
Educational background (*n* = 177)		
Baccalaureate degree (BA/BS) to RN and CNM/Graduate	111	62.7
Diploma or associate degree (AD)	38	6.5
RN to CNM/Graduate option DNP option	28	15.8
Post graduate certificate option	23	9.9
Graduate completion option	17	16.4
Other	15	7.3
Certification (*n* = 176)		
Certified nurse midwife (CNM)	173	91.1
Certified midwife (CM)	3	1.7
Certified professional midwife (CPM)	1	0.6
Direct‐entry midwife	0	0
Lay midwife	0	0
Other certification	13	7.4
Work setting (*n* = 176)		
Academic medical center	39	22.2
Public hospital/Medical facility	51	28
Private hospital/Medical facility	22	12.5
Rural clinic	11	6.3
Private practice	45	25.6
Other	8	4.6
Geographical location^a^ (*n* = 102)		
Region 1	27	15.5
Region 2	37	21.3
Region 3	27	15.5
Region 4	21	12.1
Region 5	17	9.8
Region 6	21	12.1
Region 7	24	13.7

^a^ Geographical locations including regions 1–7 were previously determined by ACNM (Regions, www.midwife.org/regions).

### 3.3 Job Responsibilities and Past Experience

Most participants reported the use of cfDNA screening in their midwifery practice (*n* = 175, 95.4%), as shown in Table 2. Patient education prior to cfDNA typically includes mention of the possibility of learning about predicted male (*n* = 146, 87.4%) or female (*n* = 147, 88%) sex. Over half of the respondents indicated that they mention the possibility of an SCA finding (60.5%, *n* = 101). Significantly less participants mentioned the possibility of discrepant fetal sex results between cfDNA screening and ultrasound images (7.8%, *n* = 13).

**TABLE 2:** Disclosure, pre‐test counseling, and experiences.

	*n*	%
Responsibilities for disclosure with prenatal screening results		
Normal cfDNA screening results	176	94.6
Normal ultrasound results	168	90.3
Sex chromosome aneuploidy (SCA)	152	81.7
Discrepant results between cfDNA screening and ultrasound imaging	117	62.9
Non‐binary genitalia	113	60.8
None	5	2.7
Potential cfDNA results participants inform patients about		
Predicted female fetal sex	147	88
Predicted male fetal sex	146	87.4
Sex chromosome aneuploidy (SCA)	101	60.5
Discrepant results between cfDNA screening and ultrasound imaging	29	17.4
None of the above	13	7.8
Prenatal screening results that participants have seen in their practice		
Normal cfDNA screening results	182	98.4
Normal ultrasound results	180	97.3
Sex chromosome aneuploidy (SCA)	120	64.9
Discrepant results between cfDNA screening and ultrasound imaging	61	33
Non‐binary genitalia	54	29.2
None	1	0.5

Participants were generally responsible for disclosing normal (risk reducing) cfDNA screening results (*n* = 176, 94.6%) and fewer, but still a majority of, participants were responsible for disclosing abnormal cfDNA screening results including SCAs (*n* = 152, 81.7%) and discrepant results between cfDNA screening and ultrasound imaging (*n* = 117, 62.9%). Regarding ultrasound results, the majority of participants reported they were responsible for disclosing normal ultrasound results (*n* = 168, 90.3%). Fewer participants reported that they were responsible for the disclosure of non‐binary genitalia (genital variations) observed on ultrasound (*n* = 113, 60.8%).

Experiences with prenatal findings suggestive of a possible fetal DSD varied. Over half of the participants reported prior experience with an SCA finding on cfDNA screening (64.9%, *n* = 120). Fewer participants reported prior experience with discrepant findings between cfDNA screening predicted fetal sex and ultrasound images (33%, *n* = 61) and non‐binary genitalia (genital variations) observed on ultrasound (29.2%, *n* = 54).

### 3.4 Terminology

**FIGURE 1:**
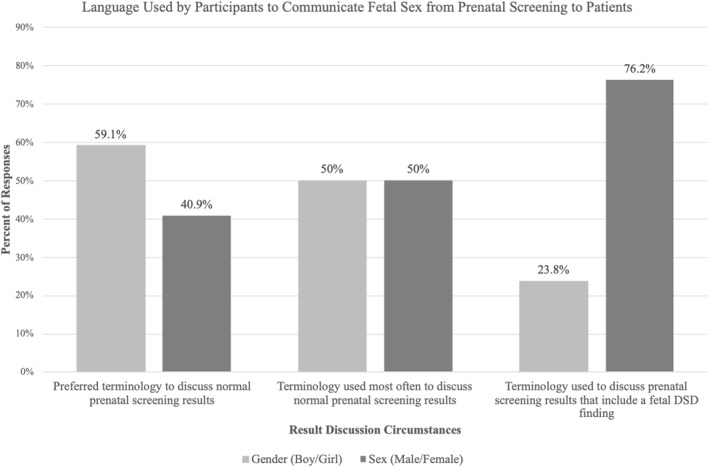
Language participants use when communicating prenatal results to patients that includes information about fetal sex (*n* = 186). The responses were combined for “Gender (Boy/Girl)” to include the terms Baby Boy, Baby Girl, Boy, Girl, and Gender. The responses were combined for “Sex (Male/Female),” to include the terms Female Sex, Male Sex, Male Baby, Female Baby, or Sex.

The majority of participants (*n* = 110, 59.1%) reported the use of gendered language (e.g., baby boy and baby girl) when communicating normal (risk‐reducing) prenatal screening results to patients. In contrast, when communicating prenatal screening results that included a suspicion of a fetal DSD, the majority of participants (*n* = 142, 76.2%) reported the use of fetal sex rather than gendered language to describe these results (Figure 1). Most participants indicated that reflecting patient language had the largest influence on the terminology that they used when talking about fetal sex (*n* = 110, 57.6%). Additional influences included work experience (*n* = 23, 12%), personal experiences (*n* = 22, 11.5%), and continuing education (*n* = 16, 8.4%).

**FIGURE 2:**
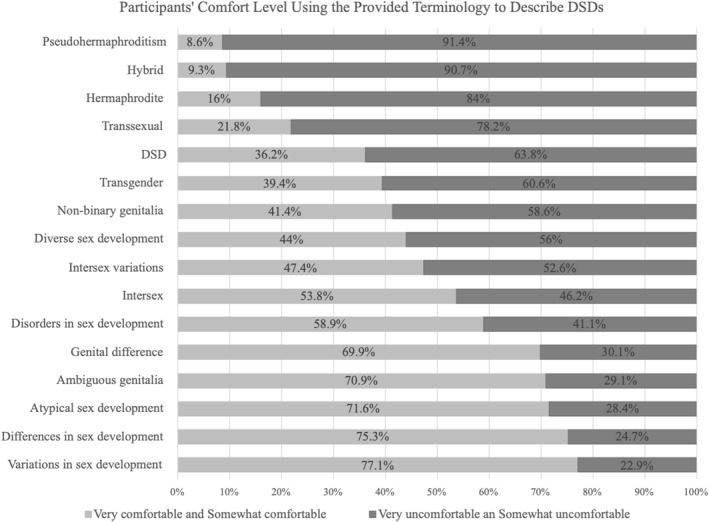
Participants comfort level using the provided terminology to describe DSD to patients. The figure combines the answer options of very and somewhat uncomfortable, and very and somewhat comfortable. The total responses for each term ranged from *N* = 172 to *N* = 176.

A majority of participants were comfortable using the following terms to discuss a possible fetal DSD with patients: “variation in sex development” (*n* = 175, 77.1%), “differences in sex development” (*n* = 174, 75%), “ambiguous genitalia” (*n* = 175, 70.9%), and “atypical sex development” (*n* = 176, 69.9%) (Figure 2). Approximately 58.9% of participants (*n* = 175) were comfortable using the term “disorders of sex development”, which was formally introduced as nomenclature referencing atypical sex development in 2006. Some participants reported comfort using terms that have been deemed inappropriate and/or outdated, including “transsexual” (*n* = 38, 21.8%) and “hermaphrodite” (*n* = 28, 16%) [32, 33, 34, 35, 36]. Participants reported that patient interactions (*n* = 48, 27.3%) and internet/media (*n* = 38, 21.6%) had the largest influences on terminology when discussing DSD.

### 3.5 Preparedness and Comfort Level

**FIGURE 3:**
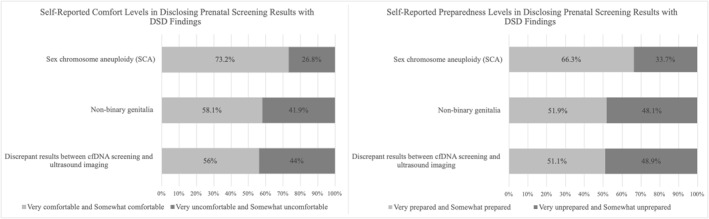
Participants' self‐reported levels of comfort and preparedness when disclosing results that included findings related to a DSD (*n* = 184 for comfort; *n* = 183 for preparedness). Answer options were combined for: Very uncomfortable and somewhat uncomfortable, very comfortable and somewhat comfortable, very unprepared and somewhat unprepared, and very prepared and somewhat prepared.

Almost half of the participants (48.9%, *n* = 89) reported that they felt unprepared to discuss discrepant fetal sex findings between cfDNA and ultrasound (Figure 3). Additionally, participants reported that they felt unprepared to discuss prenatal screening results that include non‐binary genitalia (genital variations) (44%, *n* = 81). Participants generally reported higher levels of preparedness (66.3%, *n* = 122) and comfort (73.2%, *n* = 132) when discussing SCA specifically.

**FIGURE 4:**
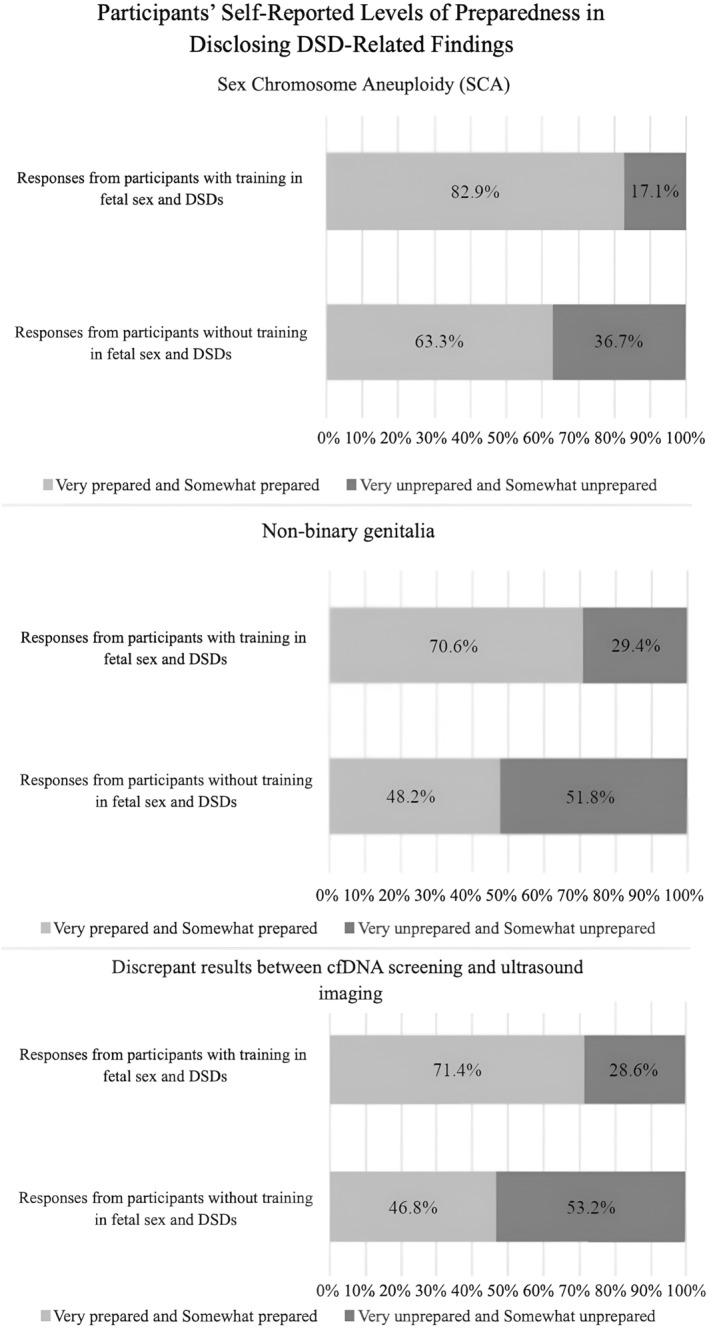
Participants' self‐reported levels of preparedness when disclosing results that included findings related to a DSD, and if participants had had training in fetal sex and DSD. The figure combines the answer options of very and somewhat prepared, and very and somewhat prepared. Responses from participants who had had training in the past (Yes) ranged from *n* = 34 to *n* = 35. Responses from participants who had not had formal training in the past (No) were *n* = 139 for the answer choices.

Participants who reported formal training on fetal sex and DSD in the past (*n* = 36, 20.3%) were statistically more likely than those without formal training (*n* = 141, 79.7%) to report feeling Very Prepared or Somewhat Prepared to disclose results that included DSD findings such as SCA (*p* = 0.027), non‐binary genitalia (genital variations) (*p* = 0.019), and discrepant results between cfDNA and ultrasound (*p* = 0.009) (Figure 4). A vast majority of respondents indicated that they would be interested in pursuing future fetal sex and DSD education (81.7%, *n* = 143).

## 4 Discussion

Since its initial introduction in 2011, cfDNA screening for fetal aneuploidy has experienced a steady rise in usage and popularity [37, 38]. Not only has this led to the integration of this prenatal screening method as a standard component of patient care, but it has also led to more frequent suspicion or diagnoses of fetal DSD [21]. Prenatal clinicians who may not have formal training in medical genetics or genetic counseling, such as CMs/CNMs, may increasingly assume responsibility for prenatal and post‐test counseling, including conveying results about a possible fetal DSD [28, 39]. Lack of attention to the terminology used when discussing fetal sex and DSD carries the risk of hindering a patient's understanding of their pregnancy, leaving patients ill‐equipped for a possible DSD finding, and perpetuating the use of inaccurate terminology [5, 40]. While this research centered on prenatal clinicians' experiences with terminology used to discuss DSD and fetal sex, it is important to highlight that the use of inaccurate terminology to discuss these findings can adversely affect the medical care of individuals within the LGBTQIA+ community. This study investigated the use of fetal sex and DSD terminology by CMs/CNMs, the factors that influence terminology choices, and their comfort and preparedness levels to discuss prenatal screening results that are atypical or unexpected. Furthermore, this study highlighted a lack of formal education among most participants regarding fetal sex and DSD, underscoring the need for specialized training in this area of healthcare.

It is important to recognize that not all individuals with a DSD identify as part of the LGBTQIA+ community, and likewise not all LGBTQIA+ individuals have a DSD. However, the use of inaccurate terminology and a lack of sensitivity when discussing DSD, sex, and gender often affects members of the intersex community. Additionally, many recorded experiences of LGBTQIA+ patients in medical settings involve insensitivity and the use of incorrect terminology [23, 24, 41, 42]. While these issues are not identical, they share commonalities in the misuse and misunderstanding of language related to sex and gender. When a DSD is suspected, it is important to engage in discussions about sex development, its inherent relationship to gender identity, and the potential for genital variation. This clarity is crucial for effective and compassionate care [18, 24, 43]. For clinicians, staying informed about terminology relevant to LGBTQIA+ individuals is key to providing inclusive, respectful, and affirming healthcare [23, 24]. Reluctance to use correct and updated terminology both perpetuates the spread of inaccurate information and contributes to the healthcare disparities faced by LGBTQIA+ individuals [23, 24, 43]. Furthermore, it reinforces a mindset among clinicians that conflates distinct concepts and continues to pathologize patients who do not fit within the traditional binary framework that is commonly taught and practiced in medical settings.

Through this research, we discovered that the majority of participating CMs/CNMs are responsible for the disclosure of prenatal screening results that could include suspicion of a fetal DSD. It was also discovered that some CMs/CNMs are comfortable using inappropriate and/or outdated terminology to explain a fetal DSD to patients. When participants were asked what they felt had the largest influence on the terminology they used to disclose prenatal screening results containing DSD related findings, they reported that it was their patients' use along with internet/media. These findings align with literature in terms of CM/CNMs previously disclosing that they are not immediately exposed to education about LGBTQIA+ care in their midwifery education. For example, Hand and Nieman (2022) and Priddle et al. (2023) found that clinicians often need to go to other sources to pursue education on LGBTQIA+ care, as this topic is often explicitly absent in their education. In our study, almost 80% of participants reported that they have not received education in the area of fetal sex and DSD. Typically, this education in the nursing and midwifery professions is often limited by the time allocated in the curriculum and left to the discretion of individual faculty members. This approach is frequently inadequate and tends to reflect cisnormative biases. As a result, such limited instruction can unintentionally reinforce binary sex assumptions and contribute to ongoing harm in clinical settings due to providers' lack of sensitivity or training [44, 45]. Educational content shaped by these statutes and deficient education in LGBTQIA+ care reinforce the practice of organizing patients and other information into strict categories, limiting the practices of medical professionals.

In this study, we confirmed that one of the largest influences on language to discuss and disclose DSD by participating CMs/CNMs is reflecting patient language. When disclosing prenatal screening results that may or may not include predicted fetal sex, the majority of respondents disclosed that they prefer to use gendered language (Boy/Girl) as opposed to sex‐based language (Male/Female). This finding is supported by past research that has discovered that prenatal clinicians are generally comfortable using the terms of sex and gender interchangeably [2, 46]. It is also worth noting that there is a recognized lack of distinction between “sex” and “gender” across many cultures and languages. In some languages, there are no direct translations or clear conceptual separations between the two terms [43, 46, 47]. This often leads to communication about someone's sex to also indicate their gender. Additionally, resistance toward individuals who do not conform to the sex‐gender binary is common. The limited recognition of gender expression further complicates discussions, especially where gender fluidity is not acknowledged [43, 46, 49]. This lack of recognition is often coupled with the absence of appropriate terminology, the criminalization of gender fluidity, and the denial of rights for trans and gender‐diverse individuals. Given these intersecting challenges, it is understandable that many cultures and countries face significant barriers to medical literacy related to sex and gender.

Being equipped with the correct knowledge about fetal sex and DSD can influence how confident and prepared a clinician feels to practice in this area of patient care. The majority of participants in this study disclosed that they felt unprepared to disclose results that may include a DSD, especially among those without previous formal training or education in this area. Similar findings have been noted in other studies that have called attention to the positive impact of relevant education [47, 48, 49, 50, 51, 52]. Discussing DSD can be complex, but it is crucial for prenatal clinicians to feel prepared and comfortable in these discussions. This is due to low levels of comfort and preparedness often being correlated with microaggressions and implicit bias in the clinical space^,^ [53, 54]. The lack of preparedness for prenatal clinicians to accurately describe sex, gender, and DSD perpetuates the marginalization of LGBTQIA+ individuals. This practice not only reinforces a sense of otherness but also contributes to discriminatory behaviors and the mistreatment of patients in clinical settings. Moreover, healthcare professionals who are inadequately informed about LGBTQIA+‐specific health issues often lack a sufficient understanding of these patients' lives, which limits their ability to conduct accurate health assessments and provide appropriate care recommendations [23, 24]. As a result, many individuals from this community report feeling responsible for educating their providers about their identities and unique healthcare needs [10, 41, 42]. Patients from the LGBTQIA+ community also frequently report negative experiences in medical settings, often due to insensitive treatment and poor communication [10, 43, 55]. For example, a study by Sharek and Sheerin et al. (2015) [56] found that 59% of LGBTQIA+ respondents believed that clinicians lacked adequate knowledge about their care. These challenges can result in heightened frustration, hesitation to seek medical care, exhaustion from repeatedly searching for knowledgeable providers, and even discontinuation of care after it has been initiated. While this research underscores the necessity for further education in fetal sex and DSD, it also demonstrates that providing such education positively influences prenatal clinicians' preparedness levels in discussing fetal sex and DSD. Results from this study could help tailor educational resources for CMs/CNMs and other healthcare professionals to enhance their understanding of fetal sex and DSD related findings. Ideally, this information will lead to more informed care and, in turn, will translate into improved patient experiences.

The increased uptake of cfDNA screening followed by the increased detection of DSD prenatally supports why both genetics and non‐genetics prenatal clinicians need to be ready for the disclosure of these results. In the future, it would be valuable to offer tailored educational resources to CMs/CNMs and evaluate their enhanced understanding, preparedness, and comfort levels regarding fetal sex and DS. Additionally, while information was collected about the current practice locations of CMs/CNMs, future research could explore how geographic location influences the practice of CMs/CNMs, including factors such as where they grew up and received their midwifery education, which were not explored in this study. Furthermore, participants were not asked about their access to or proximity to specialized clinics for DSD or intersex conditions, which could impact their practice. In addition to outlining future research directions, several limitations were identified in this study. There was inherent ascertainment bias due to voluntary participation, potentially skewing results toward those with a particular interest in fetal sex and DSD. Moreover, while the demographic profile generally reflected that of the CM/CNM profession, there was limited gender diversity, predominantly comprising cisgender females, which possibly skewed the responses to mainly reflect the experiences of this subgroup.

## 5 Conclusion

CMs/CNMs are increasingly sought after clinicians with a holistic approach to care, offering vital medical services and emotional support before, during, and after a pregnancy. One aspect of obstetrics care for which CMs/CNMs play a crucial role is the provision of cfDNA screening pre‐test counseling and result disclosure, including when there is suspicion of a possible fetal DSD. This study explored CM/CNM's experiences with cfDNA screening, including the terminology used when discussing fetal sex with pregnant patients, the factors that influence terminology choices surrounding fetal sex, and the comfort and preparedness of CMs/CNMs when there is suspicion of a fetal DSD. Findings reveal that while CMs/CNMs are tasked with conveying prenatal screening results, they often use gendered language and when discussing concerns for a fetal DSD, some CMs/CNMs continue to be comfortable with the use of outdated and/or inaccurate terminology. Further, those without DSD education or training reported less comfort and preparedness for caring for patients when there is concern for a possible fetal DSD, and most participants reported a desire for education on this topic. In summary, this study's findings underscore the need and desire for midwifery education about fetal sex and DSD to provide inclusive and informed obstetrics care, particularly surrounding the use of cfDNA screening and conversations about predicted fetal sex.

## Conflicts of Interest

Regarding conflicts of interest, one of the authors, Jeffery Dungan, MD, is now employed by Natera Inc. He was not employed at Natera Inc. while this study was conducted. This laboratory is in no way mentioned or advertised in the manuscript.

## Data Availability

The data that support the findings of this study are available from the corresponding author upon reasonable request.
